# UAV-Deployable Open-Source Sensor Nodes for Spatial and Temporal In Situ Water Quality Monitoring and Mapping

**DOI:** 10.3390/s26041158

**Published:** 2026-02-11

**Authors:** Matthew Burnett, Mohamed Abdelwahab, Joud N. Satme, Austin R. J. Downey, Gabriel Barahona Smith, Antonio Fonce, Jasim Imran

**Affiliations:** 1 Department of Mechanical Engineering, University of South Carolina, Columbia, SC 29208, USA; 2 Department of Civil Engineering, University of South Carolina, Columbia, SC 29208, USA; 3 South Carolina Governor’s School for Science and Mathematics, Hartsville, SC 29550, USA

**Keywords:** water quality monitoring, in situ sensors, UAV deployment, open-source hardware, data-driven water quality mapping, spatial interpolation (Kriging), surface water quality assessment

## Abstract

Cost efficient, spatially resolved water quality monitoring is essential for managing pollution and protecting aquatic ecosystems. This study presents a low-cost (approximately USD 200), open-source, unmanned aerial vehicle (UAV)-deployable in situ sensor node for real-time assessment of surface-water conditions. The system integrates sensors for pH, turbidity, temperature, and total dissolved solids (TDSs), with onboard data logging and real-time clock (RTC) synchronization. Bench validation of the sensor package yielded mean absolute percentage errors of 1.34% for pH, 5.23% for TDS, and 0.81% for temperature, and the device operated continuously for 42 h. Field deployment demonstrated its ability to resolve spatial gradients, with observed ranges in the tested water body of pH 6.0–6.7, turbidity 11–18 NTU, TDS 44–51 ppm, and temperature 22.8–24.6 °C. Ordinary Kriging was used to interpolate measurements and generate continuous spatial maps. The open-source, UAV-deployable design provides an accessible platform for community-scale and research-oriented water quality mapping.

## 1. Introduction

Industrial, residential, and commercial developments have resulted in significant water quality degradation, posing a severe threat to human health and aquatic ecosystems [1]. Urbanization in particular is projected to increase the delivery of nutrients, plastics, pathogens, and other pollutants to rivers throughout the 21st century [1], intensifying pressure on already stressed freshwater systems.

Traditional water quality monitoring typically relies on manual grab sampling and laboratory analysis [2]. Although these ex situ methods yield accurate and traceable results, they are labor-intensive, slow, and unable to capture real-time fluctuations in water quality. Their limited spatial and temporal resolution hinders rapid detection of transient contamination, reducing effectiveness in environmental decision-making [3]. Moreover, the logistics of sample collection and transport can introduce contamination risks that compromise accuracy, and sparse sampling grids make it challenging to characterize dynamic aquatic environments such as urban runoff channels, stormwater creeks, and agricultural reservoirs [4]. Recent review articles emphasize that many monitoring programs still operate with low-frequency sampling networks that are poorly suited to capturing short-duration pollution events or diurnal variability [3,5].

In situ sensing provides an alternative pathway for continuous, real-time water quality monitoring directly at the source [6]. By deploying sensors within the water column, immediate data acquisition enables early detection of contamination and improved understanding of short-term variability in physicochemical conditions. A wide range of in situ sensing technologies are now available, including optical and electrochemical sensors for temperature, conductivity, pH, turbidity, and selected ions or contaminants [7]. Continuous in situ measurements also support regulatory workflows by generating high-resolution datasets that can be benchmarked against applicable standards and used to constrain models [7,8,9].

Low-cost microcontroller-based platforms have further accelerated this trend. For example, several studies have demonstrated Arduino-based sensor systems for monitoring temperature, turbidity, and total dissolved solids (TDSs) in drinking water and surface waters [10,11]. Other open-hardware efforts, such as the Cave Pearl data logger, have shown that inexpensive, low-power logging systems can operate reliably for long durations in harsh environments [12]. Collectively, this body of work has established that affordable in situ sensors can deliver scientifically useful data when careful calibration and validation procedures are followed [5,7,13].

Recent low-cost systems increasingly couple multi-parameter probes with embedded microcontrollers and Internet of Things (IoT) connectivity, enabling unattended monitoring. For example, Bogdan et al. presented a low-cost IoT water quality system integrating pH, TDSs, turbidity, and temperature sensing with a mobile application workflow, targeting resource-constrained rural deployments [14]. Georgantas et al. reported an integrated low-cost monitoring station that uses LoRa telemetry to transmit reservoir pH/TDS/temperature measurements to a web information system, emphasizing open-source software for data capture and visualization [15]. Domain-specific embedded monitors have also been demonstrated, such as a microcontroller-enabled system for assessing jute-retting water quality parameters with a reported low cost [16]. Web + IoT architectures that stream pH/TDS data from NodeMCU devices into database-backed dashboards further illustrate the shift toward community-facing, low-cost monitoring platforms [17]. While most low-cost IoT stations are designed for fixed installations (e.g., tanks, channels, ponds, or reservoirs), they provide a useful baseline for sensor selection, calibration practices, and telemetry design that can be adapted to mobile deployment strategies such as UAV-enabled placement and retrieval [14,15].

Unmanned aerial vehicles (UAVs), or drones, further extend these capabilities by providing rapid, flexible deployment for environmental sensing. UAVs can access hazardous or remote sites, reduce fieldwork costs, and capture high-resolution data efficiently. Early work by Ore et al. demonstrated autonomous aerial water sampling [2]. Subsequent studies have used UAVs to sample or sense water quality in challenging environments, including mine pit lakes [18] and volcanic crater lakes [19]. Koparan et al. developed a UAV-mounted system for autonomous in situ measurement of non-contaminant water quality indicators [20]. More recently, UAV-assisted sensing platforms have been evaluated for accuracy, operational robustness, and spatial coverage in inland and urban water bodies [21,22].

In the UAV water quality literature, it is helpful to distinguish between UAV-assisted and UAV-deployed workflows. UAV-assisted systems primarily use the aircraft as a carrier for short-duration in situ probing or for collecting discrete samples that are subsequently analyzed (onboard or in the laboratory); recent work includes UAV-assisted autonomous sampling device development intended to reduce field exposure and increase sampling reach [21]. In contrast, UAV-deployed systems release a sensor node (or sampler) into the water body, where it remains to log data autonomously for later retrieval, combining the access advantages of UAVs with the persistence of fixed in situ stations. This distinction matters operationally: UAV-assisted approaches prioritize rapid spatial coverage and sample acquisition, whereas UAV-deployed approaches prioritize temporal persistence and the ability to generate time-resolved datasets at multiple spatial locations without maintaining a dense fixed infrastructure.

In parallel, there has been a growing emphasis on open-source and low-cost monitoring platforms to broaden participation in aquatic monitoring. Open hardware initiatives have produced low-cost turbidity sensors suitable for river networks [13], open-source data loggers [12], and integrated water quality monitoring systems that can be assembled with commodity components [10,11]. However, many UAV-based sensing and sampling systems described in the literature either rely on proprietary hardware, focus on single-parameter sensing, or are not documented in an open-source manner, limiting accessibility and adaptability for community-scale deployments [18,20].

Within this landscape, there remains a need for UAV-deployable water quality sensing systems that are low-cost, fully documented as open-source hardware and software, and capable of generating spatially explicit datasets suitable for modern geostatistical mapping approaches.

This study addresses this gap by presenting a low-cost, open-source UAV-deployable in situ sensor node designed for spatial and temporal water quality monitoring. The system combines calibrated sensors with a waterproof housing, autonomous data logging, and power management, all optimized for UAV-based deployment. Spatial interpolation using Kriging is employed to estimate unsampled values, enabling high-resolution mapping from sparse measurements. The contributions of this work are threefold: (1) development and open dissemination of a UAV-deployable water quality sensor node that integrates pH, turbidity, temperature, and TDS sensing; (2) demonstration of Kriging-based spatial mapping of a surface water body using data collected by the platform; and (3) a 42 h temporal analysis to assess stability and performance under real-world conditions.

## 2. Materials and Methods

This section outlines the developed sensor package, the drone deployment mechanism, and the spatial modeling technique used for spatial interpolation. The full design, including printed circuit board (PCB) files, code, and CAD files, can be found in the public repository [23]. The sensor node discussed in this study corresponds to version 0.5.0 in the repository.

### 2.1. UAV-Deployable Sensor Node

**Figure 1:**
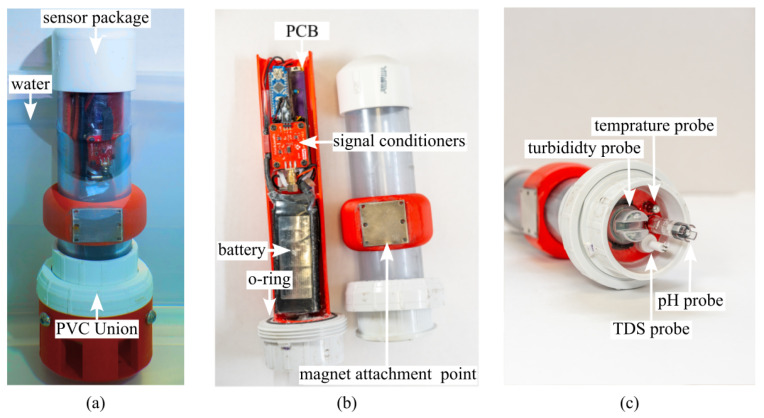
The developed UAV-deployable in situ water quality sensor package, showing (**a**) main sensor assembly in a bucket of water; (**b**) sensor probe hardware; and (**c**) sensor suite on the assembled sensor.

The sensor package is shown in Figure 1. A transparent polyvinyl chloride (PVC) tube with a 60.32 mm outer diameter (2 inch nominal) is used as the primary housing component. The protective housing hosts the components of the sensor package, including its sensors, embedded controllers, and the battery. A non-threaded union was glued to the PVC tube, as shown in Figure 1a. This union serves as the connecting link to the sensor package cap, which holds the sensor probes. The union contains an O-ring, which, when grease is applied and the thread fastened, becomes watertight. A layer of resin was applied to the surface of the cap, as shown in Figure 1c. The magnetic attachment point shown in Figure 1c doubles as a buoy to provide additional buoyancy. Given the delicate nature of the sensor suite, particularly the glass pH probe, a protective cap was integrated into the design, as seen in Figure 1a. This cap serves as an impact-resistant barrier in shallow or debris-ridden water.

The enclosure was designed to achieve IPX8 waterproofing standards, ensuring complete protection against continuous submersion [24]. The only potential entry point is the non-threaded PVC union, which employs an O-ring seal greased before tightening to maintain a watertight barrier. During validation, the fully assembled package remained submerged for 42 h without evidence of water or particulate ingress, confirming enclosure integrity.

The sensor package is relatively compact at a weight of 1.12 kg and a length of approximately 36 cm. The battery was sized to last for an ideal duration of 48 h, though environmental conditions, battery health, and sampling rate can affect operational lifetime. The detailed specifications are listed in Table 1, and the physical configuration is illustrated in Figure 1.

**Figure 2:**
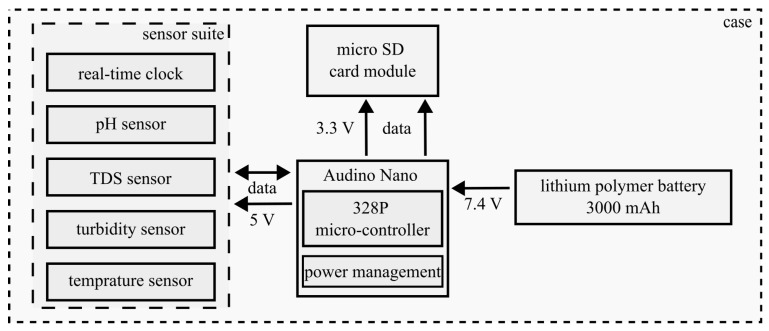
Block diagram displaying the internal components of the sensor package.

For assessing pH, an Atlas Scientific pH meter was used [20]. This analog pH sensor measures pH and consumes 15 mW, with the analog signal digitized through an onboard analog-to-digital converter (ADC) before microcontroller processing. The turbidity sensor (Keystudio V1.0) quantifies scattered light from suspended particles such as silt, sediment, and algae [10], while the total dissolved solids (TDS) sensor (Keystudio V1.0) consumes 30 mW and measures 0–1000 parts per million (ppm) by converting electrical conductivity into concentration [11]. The DS18B20 temperature sensor provides a range of −55 °C to 125 °C with ±0.5 °C accuracy between −10 °C and +85 °C [11]. These components and their signal-processing pathways are summarized in the system-level block diagram in Figure 2, which highlights the interconnections between sensing modules, the controller, the secure digital (SD) card data logger, and the real-time clock (RTC).

All sensors were factory-calibrated and subsequently verified following manufacturer-recommended procedures using supplied calibration solutions. The pH probe was three-point calibrated using pH 4.0, 7.0, and 10.0 buffers; the turbidity sensor was calibrated with 0 nephelometric turbidity units (NTUs) and 800 NTU solutions; and the TDS probe was calibrated using a 650 ppm potassium chloride standard. For TDS readings, temperature compensation was applied. The DS18B20 temperature sensor was cross-checked against duplicate thermocouples to confirm nominal ±0.5 °C accuracy. Sensor selection emphasized open-source compatibility, low power consumption, and adequate precision for surface-water studies, balancing affordability and reliability for UAV-based monitoring applications.

The firmware algorithm includes safeguards against deployment failure (Algorithm 1). A status light-emitting diode (LED) blinks to signal initialization errors or nominal operation. To mitigate SD card corruption related to low voltage, logging halts below 3.0 V, and the package then shuts down safely.
**Algorithm 1:** Sensor package data collection mission algorithm breakdown
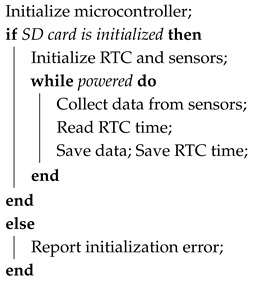


**Figure 3:**
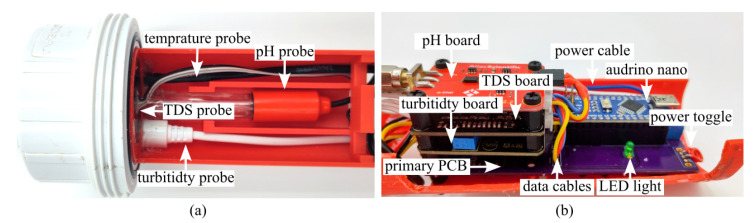
Hardware of the UAV-deployable sensor package: (**a**) top view of the probes at the bottom of the sensor; (**b**) side view of the electronics at the top of the sensor.

Figure 3 shows the electronics layout. The data collection boards for the pH, TDS, and turbidity sensors are stacked on the main printed circuit board (PCB), which also distributes power. The Arduino Nano (ATmega328P) serves as the microcontroller platform, operating at 5 V with onboard regulation and a compact footprint.

### 2.2. UAV Platform for Sensor Node Deployment

Unmanned aerial vehicles (UAVs) offer a versatile and efficient platform for deploying aquatic sensor nodes, particularly in environments that are difficult, hazardous, or costly to access manually [18]. Their ability to position instruments precisely enables high-resolution spatiotemporal data collection. Integrating the in situ sensor package with a UAV platform allows rapid, repeatable measurements across water bodies. Multi-rotor hexacopters are particularly suited to this application due to payload stability and vertical take-off and landing capabilities [20].

**Figure 4:**
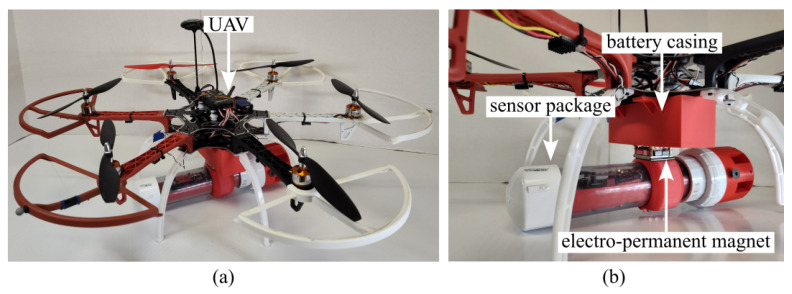
UAV used for sensor deployment showing (**a**) the complete UAV-deployable sensor package and (**b**) the electro-permanent magnet (EPM) coupling system.

The deployment mechanism employs an electro-permanent magnet (EPM) coupling system (Figure 4b). The EPM provides a low-power, mechanically simple attachment requiring only a small ferromagnetic plate on the sensor body. Upon reaching the target location, the EPM polarity is toggled to release the payload, minimizing mechanical complexity and enabling rapid, reliable deployment [25].

**Figure 5:**
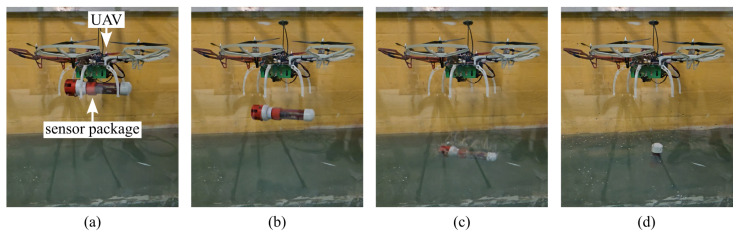
UAV deployment validation sequence: (**a**) UAV carrying the sensor package, (**b**) release via EPM coupling, (**c**) water entry, and (**d**) package self-righting and float stabilization.

Deployment testing in a controlled flume (Figure 5) demonstrated approach, release, and stabilization of the package on the water surface. Due to bottom-heavy design and distributed buoyancy, the package consistently self-righted and floated upright within one second of water contact.

### 2.3. Spatial Modeling

Kriging was selected for spatial modeling due to its capability to capture geospatial trends and provide statistically optimal interpolation from limited observation points [26,27]. The performance of a Kriging model depends primarily on the choice of variogram, which characterizes spatial correlation between measured locations. The literature studying variograms in aquatic domains indicates that the spherical model effectively represents the spatial distribution of solutes and physicochemical properties in surface and groundwater bodies [27]. Accordingly, the spherical variogram was adopted.

Kriging has demonstrated strong performance for mapping environmental and hydrological parameters, often outperforming conventional interpolation methods and remaining competitive with data-driven approaches for sparse aquatic datasets [26,28].

Equation (1) defines the spherical variogram model used to describe spatial dependence between sampling locations,

(1) $$\gamma \left(h\right)=C_{0}+C\left[1.5\left(\frac{h}{a}\right)-0.5{\left(\frac{h}{a}\right)}^{3}\right],$$

where the nugget term $C_{0}$ represents microscale variability and measurement noise, the structural variance *C* defines the magnitude of spatial variability, and the range parameter *a* specifies the distance beyond which observations are assumed to be uncorrelated. In this work, variogram parameters were estimated automatically using the PyKrige library based on the available sampling points and were not manually tuned. Given the limited number of samples, variogram fitting is sensitive to individual observations, and small changes in sampling configuration can lead to noticeable variation in estimated parameters. Accordingly, these parameters are interpreted as effective values that support spatial trend visualization rather than statistically robust or physically optimal estimates.

With larger datasets, variogram fitting would yield statistically robust parameters that can be physically interpreted, providing insight into characteristic spatial correlation lengths, dominant mixing scales, and heterogeneity of water quality processes within the monitored water body [29].

Equation (2) represents the ordinary Kriging assumption that the observed variable can be decomposed into a deterministic mean component and a spatially correlated stochastic residual,

(2) $$Z\left(s_{i}\right)=m\left(s_{i}\right)+e\left(s_{i}\right),$$

where, under the ordinary Kriging framework applied here, the mean term $m(s_{i})$ is assumed constant but unknown across the domain, while spatial structure is captured entirely by the residual component $e(s_{i})$.

Equation (3) expresses the Kriging estimator used to predict values at unsampled locations as a weighted linear combination of observed measurements,

(3) $$Z\left(x\right)=\sum_{k=0}^{K}\beta_{k}f_{k}\left(x\right)+\epsilon \left(x\right),$$

where the weights $\beta_{k}$ are determined by solving the Kriging system such that the estimator is unbiased and the estimation variance is minimized.

### 2.4. Experimental Design

A benchtop validation verified accuracy and reliability of the pH, TDS, turbidity, and temperature sensors prior to field deployment. Each package sensor was cross-compared against VIVOSUN industrial-grade reference sensors under stable laboratory conditions. During validation, both systems were immersed in identical test solutions with known concentrations and continuously monitored to eliminate transient fluctuations.

**Figure 6:**
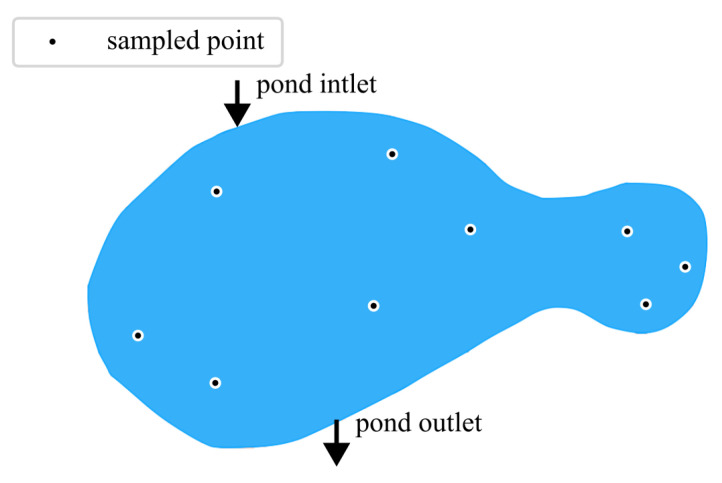
Sampling locations within the stationary testing pond used for field validation.

To demonstrate spatial monitoring capabilities, the UAV-deployable sensor package was tested in a semi-stationary pond located at the A.C. Moore Garden, University of South Carolina. The pond provided a controlled but environmentally realistic site characterized by still water with localized vegetative growth, mild organic loading, and partial canopy cover. Nine sampling points were selected to capture representative conditions across the pond (Figure 6). Each site was sampled for three minutes to account for sensor stabilization and to mitigate local turbulence effects during probe placement. While sufficient for validating the sensor, it is noted that the small sample size limits spatial resolution and interpolation reliability.

**Figure 7:**
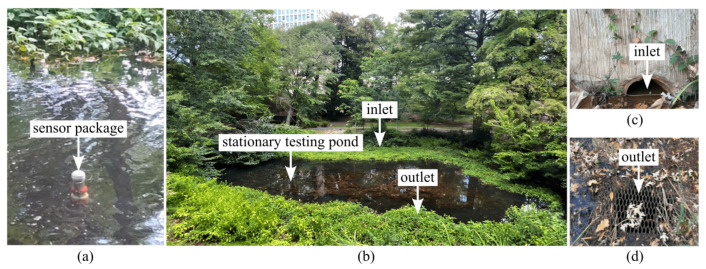
Sensor package deployment during pond testing: (**a**) the package floating on the water surface, (**b**) overview of the stationary pond, (**c**) inlet region, and (**d**) outlet region.

The site layout and representative images of the deployment are presented in Figure 7, showing the UAV-deployed package and features such as the inlet and outlet zones.

**Figure 8:**
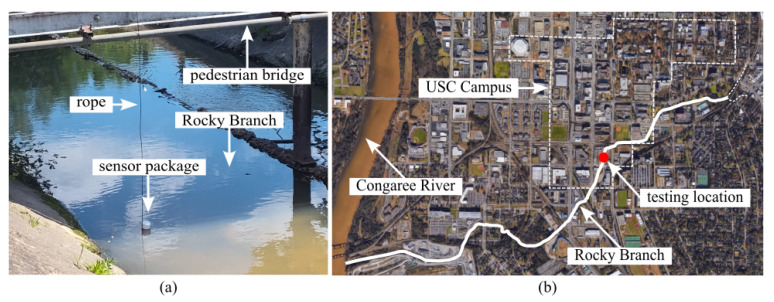
Extended 42 h test deployment at Rocky Branch Creek: (**a**) sensor package location within the creek and (**b**) the connected stormwater system.

To assess durability and long-term performance, the sensor package was deployed in Rocky Branch Creek, a municipal stormwater channel in Columbia, South Carolina, for a 42 h continuous monitoring period (Figure 8). The package was tethered in position to ensure consistent sampling depth and avoid downstream drift.

## 3. Results

### 3.1. Benchtop Validation

The Atlas Scientific pH probe, following a three-point calibration, showed excellent consistency with the reference meter and the manufacturer-provided solutions. The TDS and turbidity sensors demonstrated linear responses across the tested range. The DS18B20 temperature probe exhibited high stability, matching Type-K thermocouple readings within ±0.5 °C.

Table 2 summarizes the quantitative comparison, with mean absolute percentage errors of 1.34% for pH, 5.23% for TDS, and 0.81% for temperature. No measurable noise was observed in the ADC process, indicating effective onboard signal conditioning.

### 3.2. Water Quality Mapping

**Figure 9:**
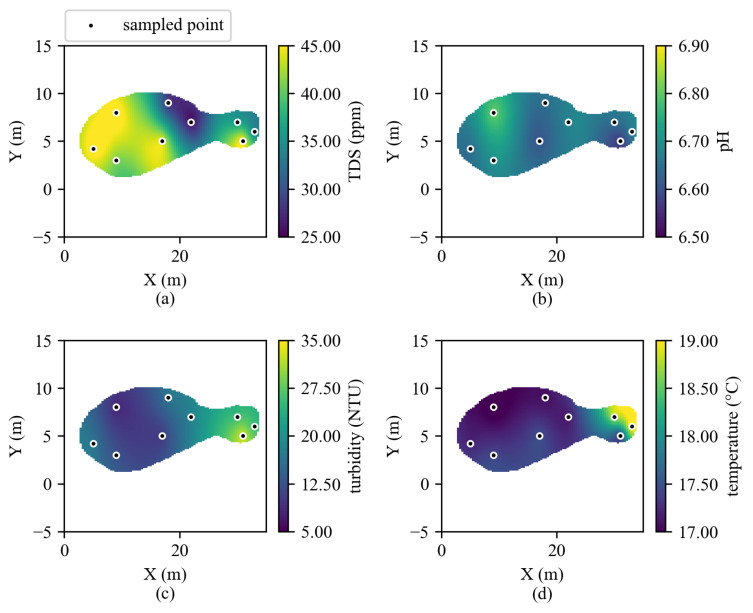
Kriging results from on-site testing for water quality mapping showing (**a**) TDS, (**b**) pH, (**c**) turbidity, and (**d**) temperature.

Using the Kriging model described previously, spatial interpolation maps were generated for pH, TDS, turbidity, and temperature (Figure 9). Distinct but modest spatial gradients were observed. TDS is elevated toward the left-central region, decreasing toward edges. The pH distribution increases toward the narrow right-hand section, indicating slightly more alkaline conditions near the outflow. Turbidity and temperature both peak along the right margin, likely influenced by greater sunlight exposure and minor inflow agitation, while the shaded left region remained cooler and less turbid.

These patterns reflect small-scale variability typical of shallow, semi-stagnant ponds, where solar heating, organic matter, and localized water movement create measurable gradients. In practical terms for South Carolina waters, these maps can be read alongside the Class Freshwater standards (R.61–68): pH 6.0–8.5 and turbidity not to exceed 50 NTU for streams/rivers (25 NTU for lakes) [30]. For TDS, neither SC nor EPA set a general ambient numeric criterion; therefore, the WHO drinking-water aesthetic threshold of 600 mg/L is referenced as an interpretive benchmark only (observed values are two orders of magnitude lower) [31]. Where national context is useful, EPA recommends pH within approximately 6.5–9.0 for freshwater aquatic life protection [32]. The mapping results are presented as a proof-of-concept in a semi-stationary pond with nine sampling locations; thus, the effective spatial resolution is constrained by sampling density and geolocation uncertainty. In higher-flow or wind-driven conditions, tethering/anchoring or position tracking would be needed to maintain association between measurements and coordinates.

### 3.3. Temporal Monitoring

**Figure 10:**
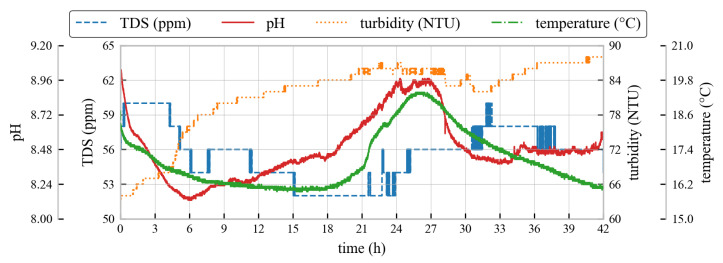
Recorded water quality parameters (temperature, pH, turbidity, and TDS) during the 42 h continuous monitoring period.

Over the 42 h test duration, the system continuously logged temperature, pH, turbidity, and TDS (Figure 10). Clear diurnal cycles were observed in temperature and pH, attributed to daytime solar heating and nighttime cooling, as well as biological respiration and photosynthesis. Turbidity and TDS exhibited episodic fluctuations, likely linked to runoff pulses or sediment resuspension events. No direct hydrological measurements were available to confirm if runoff was the cause of the observed episodic fluctuations. Despite environmental variability, power operation and logging remained stable over the full 42 h period.

**Figure 11:**
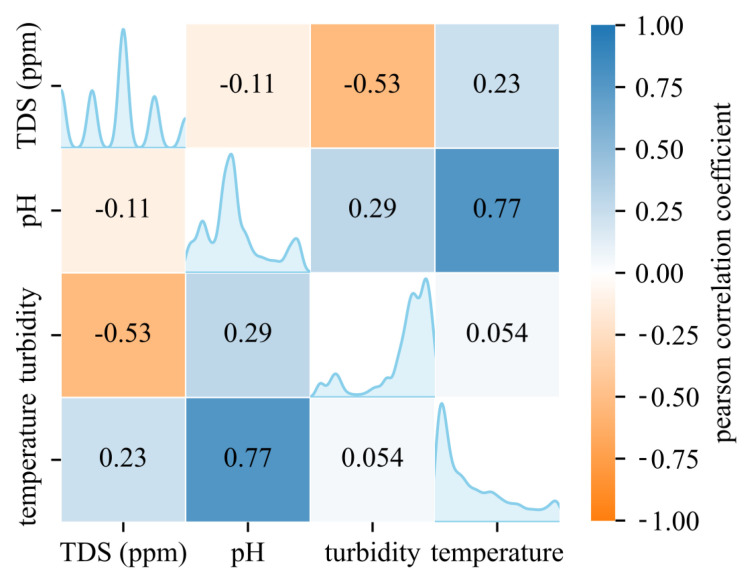
Pearson correlation coefficient matrix showing relationships between monitored water quality parameters during the extended field test.

To explore interactions among parameters, a Pearson correlation coefficient matrix was constructed (Figure 11). A strong positive correlation between temperature and pH reflects thermally coupled chemical and biological processes, while a mild inverse relationship between TDS and turbidity suggests particle–solute dynamics under variable flow.

## 4. Conclusions

This study demonstrated a low-cost (approximately USD 200), open-source, UAV-deployable in situ sensor package for surface-water monitoring, integrated with ordinary Kriging for spatial mapping using PyKrige. Laboratory validation against industrial references yielded mean absolute percentage errors of 1.34% for pH, 5.23% for TDS, and 0.81% for temperature, confirming measurement reliability within stated tolerances. While not formally certified, the sensor package reached standards comparable to IPX8 waterproofing. The enclosure achieved 42 h of continuous submersion without ingress, and uninterrupted field operation for 42 h validated power management for multi-day deployments.

Field trials produced spatially coherent maps of pH, TDSs, turbidity, and temperature that revealed fine-scale gradients consistent with localized heating, algal presence, and organic loading. For interpretive context, measured ranges are discussed relative to *South Carolina* surface-water standards for freshwaters (R.61–68: pH 6.0–8.5; turbidity ≤ 50 NTU for streams/rivers and ≤25 NTU for lakes) and EPA’s national pH guidance where applicable; for TDSs (no general ambient numeric criterion), the WHO aesthetic threshold of 600 mg/L is referenced only to aid interpretation [30,31,32]. The combined sensing and geostatistical workflow yields actionable spatial surfaces that can support hotspot identification, prioritization of follow-up sampling, and communication of spatial risk to stakeholders.

**Future work:** Planned improvements include integrating a Global Positioning System (GPS) module into the embedded system to provide more accurate coordinates, compensate for aquatic drift, and improve spatial mapping; integrating photovoltaic (PV) solar panels to extend operating time during long deployments; adding radio communications for real-time data streaming; and optimizing the package weight to reduce UAV payload requirements.

## Figures and Tables

**Table 1:** Device specifications of the UAV-deployable sensor node.

Parameter	Specification
Length	35.56 cm
Max diameter	9.2 cm
Weight	1.12 kg
Max sample rate	1 s·s^−1^
Battery capacity	3000 mAh
Battery life	42 h
pH accuracy	±0.1
TDS accuracy	±10%
Temperature sensor accuracy	±0.5 °C
Turbidity accuracy	±5%

**Table 2:** Mean absolute percentage error (MAPE) between the sensor package and reference sensors.

Parameter	Sensor Value	Reference Value	Reference Accuracy	MAPE
pH	6.62	6.71	±0.1	1.34%
TDS (ppm)	181	172	±5%	5.23%
Temperature (°C)	25.0	24.8	±1	0.81%

## Data Availability

Design files, firmware, and documentation are available in the public repository [23].

## Abbreviations

The following abbreviations are used in this manuscript:

UAV	Unmanned Aerial Vehicle
PVC	Polyvinyl Chloride
TDS	Total Dissolved Solids
NTU	Nephelometric Turbidity Units
ppm	Parts Per Million
GPS	Global Positioning System
EPM	Electro-Permanent Magnet
RTC	Real-Time Clock
PCB	Printed Circuit Board
LED	Light-Emitting Diode
SD	Secure Digital (memory card)
WHO	World Health Organization
UN	United Nations
SDG	Sustainable Development Goal
MAPE	Mean Absolute Percentage Error
